# Recruitment and Patch Establishment by Seed in the Seagrass *Posidonia oceanica*: Importance and Conservation Implications

**DOI:** 10.3389/fpls.2017.01067

**Published:** 2017-06-16

**Authors:** Elena Balestri, Flavia Vallerini, Claudio Lardicci

**Affiliations:** Department of Biology, University of PisaPisa, Italy

**Keywords:** seagrass, meadow conservation, global change, sexual recruitment, seed dispersal, *Posidonia*

## Abstract

Seagrasses are declining globally, and deeper understanding is needed on the recruitment potential and distribution of new populations for many threatened species to support conservation planning in the face of climate change. Recruitment of *Posidonia oceanica*, a threatened seagrass endemic to the Mediterranean, has long been considered rare due to infrequent flowering, but mounting evidence demonstrates that the species is responding to a changing climate through greater reproductive effort. Due to the fragmentary information on recruit occurrence and distribution, little is known about reproductive success in the species and its contribution to persistence. We assembled *P. oceanica* recruitment data from published and unpublished sources, including our own, to examine the frequency and extent of recruitment events (establishment of seedlings in a site), seedling growth potential and habitat characteristics at recruitment sites. Results show that at least one recruitment event has occurred about every 3 years, and 18 localities were colonized at least one time since the first seedling record in 1986. Notably, consistently high seedling inputs were observed in four localities of the Western Mediterranean. Seedlings established mainly on unoccupied substrate areas along the coasts of islands, in sheltered sites and at shallower depths (<3 m) than the upper limit of adjacent *P. oceanica* meadows. Seedling establishment occurred more frequently on rocky than on sandy substrate, and rarely on dead “matte” or meadows of the seagrass *Cymodocea nodosa*. The chance of colonization success on rock was two times higher than on sand. Our 11 years of observations have allowed for the first time the documentation of the formation and development of patches by *P. oceanica* seed. These findings contradict the historical assumption that sexual recruitment is rare and usually unsuccessful for *P. oceanica*, and highlight the potential importance of recruitment for the long-term persistence and adaptation of the species to sea level rise predicted in the next century in the Mediterranean. Unfortunately, management actions have mainly focused on established meadows, ignoring the presence of recruits in outside areas. Therefore, it will be useful to identify and consider regeneration sites in designing future management strategies to improve seagrass conservation effectiveness.

## Introduction

Seagrasses form highly productive ecosystems which are amongst the most valuable around the globe (Costanza et al., 1998) but have declined worldwide due to anthropogenic pressures and natural disturbances (Short et al., 2011; Unsworth et al., 2015). Predicted climate-induced environmental alterations (IPCC, 2007) are expected to exacerbate this decline (Short et al., 2011; Saunders et al., 2013), and in many countries substantial conservation efforts are underway to prevent further loss of vegetative material and ensure habitat quality standards for plant growth (Kenworthy et al., 2006). To date limited attention has formally been paid to recruitment by seed, which is the most vulnerable phase of seagrass life cycle, and rarely has sexual material been used in the context of restoration (van Katwijk et al., 2016). This is mainly because of the historical paradigm that sexual reproduction contributes only marginally to the maintenance and growth of populations. However, there is growing recognition that the long-term persistence of species depends to some extent on sexual reproduction, as even low rates of recruitment may provide evolutionary potential and increased resistance/resilience to environmental alterations (Reusch et al., 2005; Ehlers et al., 2008; Reynolds et al., 2012; Kendrick et al., 2016). Recruitment by seed also plays a role in colonizing new habitats or recolonizing following local extinction, and contributes to patch expansion in some species (Orth et al., 2006; van Dijk et al., 2009; Kendrick et al., 2012; McMahon et al., 2014; Furman et al., 2015; Sherman et al., 2016; Smith et al., 2016). Some authors argue that the protection of areas that are sources of sexual propagules or that receive high seed inputs, or both, should be a conservation priority (Kenworthy et al., 2006; Orth et al., 2006). Unfortunately, knowledge of the distribution of seeds and seedlings, which is essential to prioritize conservation locations, is scarce and mostly based on casual observations for species that produce positively buoyant fruits (Ruiz-Montoya et al., 2015), possibly because of the inherent difficulty in predicting where seeds will settle.

This is certainly the case for the slow-growing seagrass endemic to the Mediterranean, *Posidonia oceanica* (L.) Delile, that forms extensive meadows from 0 to 40 m depth. This species produces large (about 1 cm in length, Balestri et al., 1998a) positively buoyant fruits that may be transported 100s of kilometers from parent meadows under the influence of wind and surface currents before release of seeds (Micheli et al., 2010), a strategy that maximizes the colonization potential of new habitats. The seed is non-dormant, and germination occurs after maturation of the seed inside the fruit. Laboratory studies showed that the germination rate is generally high (>90%), and the seed remains attached to the seedling, consisting of a single shoot and few adventitious roots, for up to 2 years after germination (Balestri et al., 1998a, 2009). However, recruitment by seed has long been considered to be rare due to infrequent flowering (Buia and Mazzella, 1991), and most meadows are thought to be primarily the result of ancient stepwise colonization events with very few migrations, and a dominant clonal space occupation (Arnaud-Haond et al., 2014) typical of species with a predominant Initial Seedling Recruitment strategy (ISR; Eriksson, 1993). The presence of very old *P. oceanica* clones indicates that the species has persisted through past climate changes and large fluctuations in sea level (Arnaud-Haond et al., 2012). But during the last 50 years between 11 and 52% of the documented surface area originally occupied by the species has been lost, and many extant meadows are in regression due to the cumulative effects of local anthropogenic pressures (Telesca et al., 2015). Given the slow rate of rhizome elongation (2 cm year^-1^) and infrequent sexual reproduction, the recovery of meadows from large scale disturbances is considered irreversible at human-life time scales (Marbà and Duarte, 1998). *P. oceanica* is listed as a species of Least Concern within the International Union for the Conservation of Nature Red List of Threatened Species (IUCN, 2015) and included in Annex I of the Bern Convention (Convention on the Conservation of European Wildlife and Natural Habitats, 1979) as a protected species. The habitat has been also identified as a priority under the European Commission Habitats Directive (92/43/EEC), and in several European countries the species and/or the habitat are under specific legal protection (Boudouresque et al., 2012).

Projected increase of seawater temperature could pose further threats to extant meadows, possibly leading to functional extinction of those in regression (Marbà and Duarte, 2010; Jordà et al., 2012). This prediction, however, is associated with considerable uncertainties because of the high plasticity of the species and other factors that may interact with climatic factors (González-Correa et al., 2007). Moreover, the massive flowerings recorded over the last decades (Balestri, 2004; Diaz-Almela et al., 2006) coincident with exceptionally high summer seawater temperatures (Diaz-Almela et al., 2006) have prompted the hypothesis that the species is already reacting to changing climate through an increased allocation to reproduction, a phenomenon that has also been observed in terrestrial plants (Schauber et al., 2002). Naturally established seedlings have also been recently found in some localities, and the few studies that have monitored the fates of these seedlings have shown that most of them usually perish during their first year due to physical disturbances (Balestri et al., 1998b; Piazzi et al., 1999; Balestri and Lardicci, 2008) and nutrient stress (Balestri et al., 2009). Investigations on the spatial patterns of seedling distribution at local scale, i.e., a single locality, suggested a possible relationship among microhabitat type (substrate, algal vegetation, and microtopography), seedling establishment, and survival rates (Balestri et al., 1998b; Piazzi et al., 1999; Balestri and Lardicci, 2008; Alagna et al., 2013). However, conclusions derived from single locations cannot be extrapolated to a broad scale (i.e., whole Mediterranean). Direct evidence of patch establishment by seed and expansion is still lacking, and what constitutes a suitable habitat for regeneration of the species remains to be elucidated. Moreover, no attempt has been made to examine the spatial extent and frequency of recruitment events. This information is fundamental to fully capture the importance of sexual recruitment for the long-term persistence and spread of the species. It is also crucial to identify the presence of areas that will support new populations in the future and hence may help conservation managers to establish more effective interventions.

In this study, we reported results of field investigations on the occurrence of *P. oceanica* recruitment in different localities along the coasts of Corsica (France) and the long-term (11 years) evolution of seedling patches established in one of these localities in 2004. We also estimated the global frequency of *P. oceanica* recruitment events, the total number of recruitment sites and the geographical distribution of seedling populations across the Mediterranean by using presence records of naturally established seedlings compiled from published literature and unpublished studies, together with our own unpublished data. All available data were used to derive information on the abundance of recruits and the environmental characteristics at the recruitment sites. Particular attention has been paid to assess whether some localities consistently received high recruit inputs. Moreover, available data on the establishment success at those localities where the fate of seedlings was monitored over time were analyzed to gain insights into the habitat conditions, in particular depth and substrate type, potentially favorable to the establishment and expansion of seedling populations. Finally, we provided suggestions on improving seagrass conservation planning and management actions.

## Materials and Methods

### Field Observations of Seedlings Established in Corsica (France)

**FIGURE 1:**
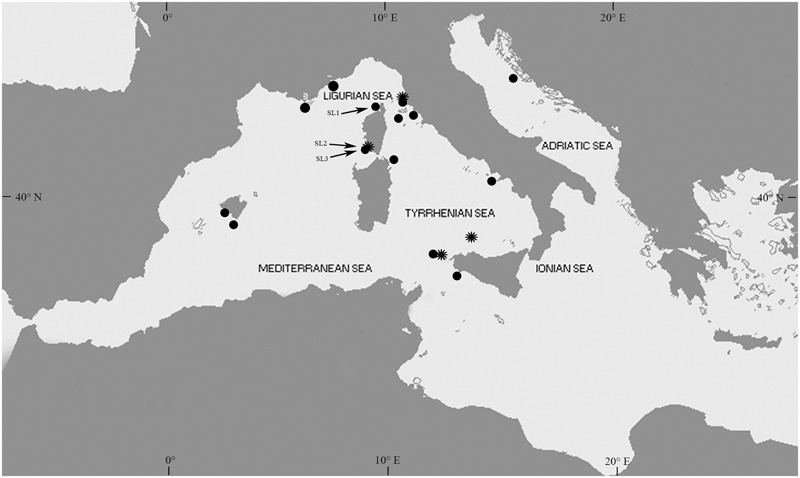
Map of the localities with *Posidonia oceanica* recruitment citations since the first *in situ* seedling record of 1986 until 2016. SL1, SL2, and SL3 indicate the three localities in Corsica (France), Saint Florent, where recruits were observed in July 2006, and Ventilegne and Tonnara where recruits were observed in July 2016. The symbol ^∗^ indicates the four localities that received consistently high recruit inputs.

In July 2006 and 2016, the presence of *P. oceanica* seedlings was recorded in three localities (**Figure 1**) along the coasts of Corsica (France): Gulf of Saint Florent (2006), Gulf of Ventilegne (2016), and Tonnara (2016). In each of the first two localities, the density of seedlings was estimated by counting the number of seedlings in 10 quadrats (50 cm × 50 cm) randomly allocated in two sites, 100s of meters apart. Only one site was available at Tonnara. Seedlings were distinguished in the field from isolated shoots established from vegetative fragments owing to the presence of the seed or seed coat still attached to the rhizome and shorter leaves (<15 cm in length). The age of seedlings was determined on the basis of the total number of leaves (plus sheaths) per shoot, and the presence and color of the seed. We defined as newly established seedling (<1 year) a single shoot with 4–10 leaves and an attached a green seed, while we defined as well-established seedling (>1 year) as a single shoot with more than 10 leaves and an attached dark seed.

To evaluate the capacity of seedlings to establish new patches and colonize new substrate, six randomly selected seedling patches (three patches on sand and three patches on rock) observed in one site of the Gulf of Ventilegne (Balestri and Lardicci, 2008) were marked at their edge and photographed in July 2005. At the start of the monitoring period, the shape of these patches was approximately circular, and their maximum width varied from 0.15 to 40 cm. To monitor their evolution, the patches were followed yearly till July 2016 by visual inspections and photographs.

### Literature Search

We searched all papers (peer-reviewed articles, gray literature and personal communications from other authors) that provide direct or indirect evidence of natural establishment of *P. oceanica* seedlings in the electronic library databases (e.g., Web of Science, Thomson Reuters, New York, NY, United States and Google, Mountain View, CA, United States). Two sets of keywords were used. The first set included words the associated with the seagrass (seagrass) or scientific name (*Posidonia oceanica*). The second set of keywords contained words associated with sexual reproduction (sexual reproduction, recruitment, seedling, or flowering). Each word in the first set was searched in combination with each word from the second set (last search date: 05/10/2016). In addition, to take into account older references that may not be available through ISI Web of Science, the reference list in each article was also scanned for any other relevant publications or unpublished observation. Delocalized citations (seedlings stranded on beaches or floating) were also examined even if not considered as successful recruitment.

From all available reports and our own unpublished data on the recruitment of *P. oceanica* by seed, we gathered information about the year of seedling observation, the country, the geographical area, the locality name and the geographical coordinates where seedlings were found. When not reported, latitude and longitude of localities were indirectly estimated from an atlas. When seedlings were found in different sites (usually 10s of meters to kilometers apart) within the same locality, the name of the sites was reported when available. When seedlings were observed on different substrate types or depths within the same site, the observations were considered as a unique recruitment record. In addition, we extracted data on the age of seedlings at the time of observation and the year of establishment, the duration of seedling observation, local environmental characteristics (coastal features, water depth, habitat and substrate type), proximity to established meadows, management status (no protection or protection) and anthropogenic pressure level at the recruitment locality. The level of pressure was considered to be moderate or high in proximity to fishing ports, urbanized or industrial areas, coasts with altered sedimentary/hydrologic regimes, river mouths or boat traffic, and low in protected marine areas or natural parks. Quantitative data on seedling abundance (mean number of seedlings m^-2^) in a given locality at the time of observation where retrieved when possible. When seedlings were present in different sites in the same locality, the relative mean abundance data were reported. Since in some cases data on seedling abundance were presented as range, the geometric mean was calculated and retained as an individual record of abundance. However, different authors used different sampling designs, sizes, or methods for determining seedling density. For some localities only the number of seedlings collected for morphological analysis or the total number of seedlings counted was available. Data on rhizome branching or production of new shoots and evidence of biotic disturbances (i.e., grazing on seedlings by fishes or sea urchins) were also retrieved when available. The search parameters were summarized in **Table 1**. Data obtained from all reports and our own observations were used to construct a database (Supplementary Table S1).

**Table 1:** Parameters and data gathered from the literature search.

Search criteria	Search parameters
*Seedling observation period*	Year and month of observation
	Establishment year of seedlings
*Location characteristics*	Country
	Region
	Exposure level (sheltered vs. exposed)
	Name of locality and sites (when available)
	Geographical coordinates (latitude N and longitude E)
	Human pressure level (high, moderate, low)
	Management measures of protection
	Presence of well-established *P. oceanica* meadows
*Habitat characteristics*	Maximum and minimum depth of seedlings
	New substrate areas, previously colonized areas (dead “matte”) or seagrass bed
	Substrate type (sandy substrate or rock substrate)
*Seedling data*	Abundance (number of seedling m^-2^ or total number of sampled seedlings at each locality/site)
	Seedling age at the time of observation
	Survival (percentage of seedlings survived in each locality/site at the end of the monitoring period when available)
	Formation of new rhizome branches (yes or not)

From all reports, we determined the global frequency of recruitment as number of years in which at least one successful event has been recorded divided per total number of years since the first seedling record. For each locality, we calculated the total number of recorded events. The total number of sites with recruits in a given year was also determined and the intensity of recruitment for each recruitment year was expressed as total number of sites in which seedlings were observed in that year. The spatial extent of recruitment was determined as total number of sites covered by recruitment since the first seedling record. The different types of substrate where recruits were found were categorized in four classes (sandy substrate, rocky substrate, dead “matte” and seagrass bed) and the total number of occurrence on each habitat class was calculated by combing data from all sites and years. When seedlings were found in the same habitat within the same site but in different years, the observations were considered as separate records. As seedlings were often present at different depths in the same site, the minimum depth (upper limit) and the maximum depth (lower limit) reached by seedlings were extracted from available data and the relative distribution frequencies analyzed to determine the average depth range of distribution of seedlings.

From available data on the abundance of seedlings at the time of observation we derived the overall mean of seedling abundance. When seedlings abundance data were available for different sites or substrate areas in a given locality, the mean seedling abundance for that locality was calculated by pooling data from the different sites or substrate areas. As the first year is considered the most critical for *P. oceanica* seedlings (Balestri et al., 1998a,b), for the purposes of this study, we considered well-established seedlings to be only those seedlings >1 year in age. To determine the chance of colonization success we thus selected those studies that monitored the fate of seedlings for at least 1 year or those that documented the presence of seedlings at least 1 year old. The total number of observations that documented the persistence of at least one seedling for more than 1 year (success) or the death/loss of all established seedlings (failure) in a given habitat type (sand vs. rock vs. dead “matte” vs. seagrass bed) and depth class (shallow vs. deep) was also determined and the relative frequencies of success/failure calculated by pooling data from all sites and years. As the type of substrate (rock vs. sand) and depth have been regarded as the main drivers of recruitment success at local scale (Piazzi et al., 1999; Badalamenti et al., 2015), the number of successes recorded for each substrate class (rock vs. sand) irrespective of depth, and for each depth class (shallow vs. deep) irrespective of substrate, was analyzed by Fisher exact *p*-test. Since only a few observations for seedlings on dead matte (4) and seagrass bed (1) were available, the relative number of success in these habitats were not analyzed.

## Results

### Field Observation of Seedlings Established in Corsica (France)

**FIGURE 2:**
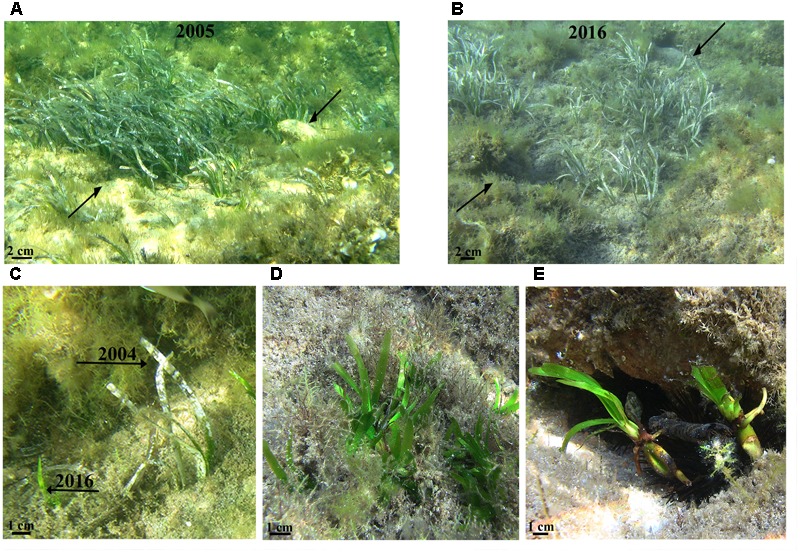
Seedlings of *P. oceanica* established in the Gulf of Ventilegne (Corsica, France). A patch of seedlings established in 2004 on a sandy substrate patch and marked (arrows) in July 2005 **(A)** and the same patch photographed after 11 years in July 2016 **(B)**. Newly established seedlings (2016s cohort) found on sandy substrate very close to old seedlings (2004s cohort) **(C)**, on rocky pools **(D)**, and on the aboral surface of sea urchins *Paracentrotus lividus* **(E)**.

Seedlings of *P. oceanica* observed in the Gulf of Saint Florent in July 2016 showed a dark seed coat still attached to the base of rhizome and the total number of leaves plus leaf scars produced by seedlings (20.3 ± 1.6 SE) was similar to that previously recorded in seedlings (22) maintained in culture for about 2 years (Caye, 1989), suggesting that they were approximately 2 years old, and thus established in the summer 2004. In one of the two selected sites, seedlings colonized an area of about 300 m^2^ (mean seedling density, 22.0 ± 1.7 m^-2^ SE) of rocky substrate covered by macro-algae, at 0.5–1.5 m depth, in proximity to the upper limit of a *P. oceanica* meadow. In the other site, isolated seedlings (1 seedling m^-2^) were established on sandy patches within a *Cymodocea nodosa* (Ucria) Ascherson bed, at 0.5–1.5 m depth. The seedlings observed in the Gulf of Ventilegne in July 2016 (**Figure 2**) were very young (<1 year) and established in the same two sites where seedlings were found in 2005. In one of these sites, newly established seedlings were located on sandy patches at a depth of 0.5–2 m (mean seedling density, 42 ± 7 m^-2^), while in the other one they were present both on sandy patches (mean seedling density, 8.1 ± 1.3 m^-2^) and on rock (mean seedling density 9.3 ± 2.6 m^-2^) at 0.4–2 m depth. Many seedlings established in 2016 were close (centimeters apart) to young plants established by seed in 2004 (**Figure 2**). Patches of seedlings (mean seedling density, 31.3 ± 7.4 m^-2^) were also found into rocky pools at very shallow depth, 0.10–0.20 m depth (**Figure 2**). Seedlings on the aboral surface of the body of sea urchins, *Paracentrotus lividus* (Lamark), were observed (**Figure 2**). Seedlings found at Tonnara (Gulf of Ventilegne) in July 2016 were also very young (<1 year) and established at very shallow depth (0.10–0.20 cm) on rocky substrate colonized by macro-algae. Only one seedling per m^2^ was found in this site (1 seedling m*^-^*^2^).

All seedling patches marked on sand in 2005 in the site of Ventilegne were still present in July 2016, but they showed a substantial change both in their form and structure. Many seedlings, especially those initially situated in the central area of the patches, had disappeared (**Figure 2**) and this loss estimated in terms of percent cover accounted for 75% (± 2.88) of the original patch size. However, the loss on sandy patches was compensated by the growth of horizontal rhizomes and production of new shoots in surviving seedlings at patch edges (**Figure 2**). Instead, only 1 seedling per patch survived on rock and the young plants generated from survived seedlings showed up to three shoots at the time of observation (July 2016).

### Frequency and Spatial/Temporal Patterns of Recruitment Events

**FIGURE 3:**
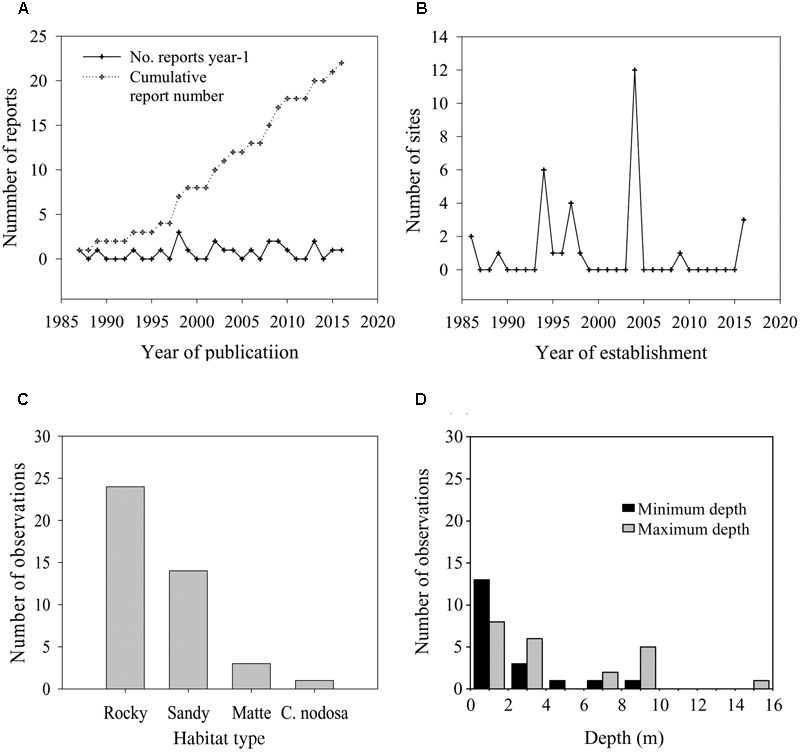
Number of reports that documented the presence of *P. oceanica* seedlings identified through the literature search amended with our own records **(A)**, total number of sites where seedlings were established in a given year since the first seedling record of 1986 **(B)**, total number of observations of seedlings on a particular habitat type **(C)** and frequency distribution of minimum colonization depth (upper depth limit) and maximum colonization depth (lower depth limit) of seedlings at recruitment sites **(D)**.

The first record of naturally established *P. oceanica* seedlings was reported in the 1987. Since this record, twenty-two citations in the scientific literature and gray literature, including personal communications from other authors and our own data, reported the presence of seedlings (**Figure 3** and Supplementary Table S1). Five of these citations documented the presence of seedlings floating or stranded on beaches. Four out the remaining citations did not provide an indication of water depth and/or substrate type at the recruitment site, and one reference did not specify the year of seedling establishment. Only seven of the total records of *in situ* seedlings have been published in international peer-reviewed journals. Recruitment events have been recorded in 18 localities from four countries, Italy, France, Croatia, and Spain (**Figure 1** and Supplementary Table S1). The latitudinal range of seedling distribution covered a relatively large band, from 37°39′ to 43°58′ N (**Figure 1**). In the Eastern Mediterranean basin, recruitment was observed only in one locality of the Central Adriatic Sea region (Dugi Otok Island, Croatia), and this was the northern extreme of the seedling distribution. The remaining events were recorded in the Western Basin; in the Balearic Sea to Tyrrhenian Sea (15 locations), and Gulf of Lyon and Ligurian Sea (three locations) regions. The southern extreme of the seedling distribution was in the Tyrrhenian Sea, Capo Feto (Sicily Island; Italy). Stranded seedlings were observed in eight localities of the Western Mediterranean and in one locality of the Eastern Mediterranean (Ionian Sea), providing evidence of a potential recruitment event in the neighborhood, but they were excluded from the analysis. In six localities (Favignana, Ustica, Gulf of Ventilegne, Gulf of Saint Florent, Livorno, and Mallorca), seedlings were found in different sites, and in total thirty sites had at least one recruitment event (**Figures 1, 3** and Supplementary Table S1). From 1986 and 2016, the presence of new recruits was noticed 10 of the years (**Figure 3**), corresponding to a frequency of about 0.33 events y^-1^, and at least one recruitment event was observed every year from 1994 to 1998. The mean number of sites colonized every year was 1.03 (± 0.04 SE), and in those years in which at least one recruitment event was observed, the number of colonized sites varied from 1 to 12 (mean, 3.20 ± 1.11 SE). The higher intensities of recruitment (**Figure 3**) were recorded in 1994 (five localities, six sites in total,) and 2004 (eight localities, 12 sites in total). Two recruitment events each at Ustica Island (in 1997 and 2009), Livorno (in 1994 and 2004) and Gulf of Ventilegne (in 2004 and 2016), and three events at Favignana Island (in 1994, 1995, and 2004; Supplementary Table S1) have been recorded.

### Habitat Type, Depth, and Colonization Success

Most localities (72%) where seedlings were found are situated along the coasts of islands (Supplementary Table S1). The presence of recruits along continental coasts was reported only in five localities, Gulf of Giens, Gulf of Juan, Livorno, Rosignano Solvay, and Follonica. Seven localities were characterized by moderate or high levels of anthropogenic pressure, while the remaining ones were located in zones with low levels of pressure such as marine protected areas and marine parks. Seedlings colonized mainly sheltered sites usually along curvilinear coasts such as bays and gulfs, or small inlets. Only three localities were situated in relatively exposed sites, Rosignano Solvay, Livorno, and Capo Feto. In all recruitment sites, seedlings were close to established meadows. Seedlings were found in previously unoccupied areas colonized by macro-algae, except that in three localities (Follonica, Rosignano Solvay and Livorno, Italy) where they were recruited within dead “matte” areas in disturbed meadows, and in one locality (Saint. Florent, Corsica), in which they were present in a *C. nodosa* bed (Supplementary Table S1). Established seedlings were also found in rocky pools in two localities, Gulf of Ventilegne (Corsica) and Pantelleria Island (Italy). In five of the 18 localities seedlings were present on two different types of substrate, sand and rock or sand and dead “matte” (Supplementary Table S1). The total number of occurrences of seedlings on rocky habitats was about twofold more than that on sandy habitats (**Figure 3**). Seedlings were found at different depths, from 0 to 15 m, but in only two locations did they reach depths below 10 m (**Figure 3** and Supplementary Table S1). The mean minimum depth of seedling distribution did not exceed 2 m (mean, 1.9 ± 0.5 m, median depth limit 1 m), while the mean maximum depth was above 5 m (mean 4.9 ± 0.89, median depth limit 3 m). Seedlings colonizing sandy substrates and dead “matte” were generally located deeper (2.9 ± 0.8–6.0 ± 1.24 than those on rock (0.9 ± 0.22–4.2 ± 1.03) or in *C. nodosa* beds (0.5–1.5 m). The deeper seedlings were observed on dead “matte” (5.6 ± 2.33–10 m). Data on seedling abundance were available for nine localities (Supplementary Table S1). Mean seedling density recorded in 2004 in the Gulf of Ventilegne (135.2 ± 24.3 m^-2^) was more than fourfold the average seedling density of all localities (20.7 ± 12.9 m^-2^).

In five localities seedlings were 1 or 2 years-old at the time of observation and hence they had survived the most critical life period. In six localities, established seedlings were monitored over time but the duration of the observational period was limited (<3 years). Only in one locality, Gulf of Ventilegne, did the observational period exceed 3 years (11 years, present study). The establishment of seedlings was successful in all monitored localities and sites as at least one seedling was still present at the end of the monitoring period. Overall, the chance of colonization success on rocky substrate was significantly higher, about twofold more than on sandy substrate (**Table 2**) irrespective of depth (*p* = 0.008). The probability of success for seedlings on dead “matte” and *C. nodosa* beds was 100% (**Table 2**) but too few observations were available to infer meaningful conclusions about the recruitment success of seedlings in these habitats. No difference was detected between shallow and deep depth classes (*p* = 0.56). Four studies reported the production of at least one new rhizome ramification (Supplementary Table S1) and only the present study documented the expansion of seedling patches. Clear evidence of damage on seedlings by herbivores was never observed (Supplementary Table S1).

**Table 2:** Chance of colonization success by *P. oceanica* seedlings: total number of successes or failures and overall frequency of success (%) of colonization classified according to (a) habitat type (substrate class) and depth class and (b) depth class alone.

	Success	Failure	Success	Failure	
Variable	Depth ≤3 m		Depth ≥3 m		Overall success (%)
(a) Substrate class					
Rock	12	1	2	0	93.3
Sand	2	3	1	2	37.5
Dead matte	1	0	3	0	100
*C. nodosa*	1	0			100
(b) Depth class					
≤3 m	16	4			84.2
>3 m	6	2			71.4

*The persistence of at least one seedling on a given substrate or depth in a site was considered as colonization success while the loss of all seedlings was considered as failure. The depth of 3 m corresponded to the median maximum depth reached by seedlings. Data are gathered from available studies that have monitored *in situ* seedling development or that reported the presence of seedlings 1 or 2 years-old at the time of observation. Data from different years and sites were pooled.*

## Discussion

The results of the present study provide insights into the frequency and success of sexual recruitment in *P. oceanica* across the Mediterranean. In particular, the observed successful establishment and expansion of patches by seeds and the frequent and consistent recruitment events recorded in some locations in the last three decades indicate that sexual reproduction could be more relevant for the species than previously thought with management/conservation implications in the face of changing climate conditions.

### Field Observation of Seedlings in Corsica (France)

Our long-term field observations of seedlings established in the Gulf of Ventilegne in 2005 allowed us to document the formation of patches by seeds and their evolution over their first decade, demonstrating that under particularly favorable conditions seedlings may persist and successfully colonize sandy substrates even at very shallow depths. The slow rate of expansion of these patches (10s of centimeters in 11 years) is in agreement with the prediction of simulation models which indicate that a new clone of *P. oceanica* may take 100 years to attain a diameter of 8 m (Kendrick et al., 2005). A previous analysis of the initial spatial distribution of seedlings at Ventilegne has shown an aggregated pattern with higher seedling density closest to the center of seedling patches and lower densities at increasing distance from the center (Balestri and Lardicci, 2008). A positive relationship between seedling density and mortality rate in the post-establishment phase also emerged from the previous study (Balestri and Lardicci, 2008). Here, the formation of gaps from mortality inside seedling patches supports the hypothesis that competition for limiting nutrients or available space occurred among some seedlings as they increased in size due to overcrowding. But, a change in the topography of sea floor due to physical disturbance, in particular the formation of erosion channels created by elevated levels of turbulence within the patches during severe storms, might have played a role. Even though high herbivore consumption rates of fruits/seeds have been reported (Balestri and Cinelli, 2003) and bite marks on leaves were found on transplanted seedlings of *P. oceanica* (Domínguez et al., 2012), herbivore pressure did not appear to be a threat for early life stages. However, our observations revealed that the sea urchin *P. lividus*, one of the major consumers of *P. oceanica* fruits, may cover itself with detached seedlings. Further studies would be necessary to establish whether, and if so to what extent, this cryptic covering behavior removing seedlings from rocky substrate, may reduce the success of establishment.

### Frequency and Spatial/Temporal Patterns of Recruitment Events

Previous studies have shown that *P. oceanica* meadows have flowered on average every 5 years over the Mediterranean Sea in the last three decades (Diaz-Almela et al., 2007). Our analysis revealed that at least one recruitment event has occurred approximately every 3 years since the first seedling record in 1986. Clearly, this is a conservative estimate likely underestimating the actual frequency of recruitment, being based on occasional records rather than on systematic research. Only one recruitment locality (Dugi Otok Island, Croatia) was in the Eastern Mediterranean, and the remaining localities (17) were in the Western Mediterranean. This distribution might reflect either an unequal investment in research with a high density of observational efforts concentrated in the western sector and a paucity of studies on the eastern sector of the basin, or a different flowering frequency/intensity of meadows in the two sectors (Diaz-Almela et al., 2007), or a combination of both. The higher intensities of recruitment events, in terms of number of colonized sites, followed the massive flowerings of 1993 and 2003 (Diaz-Almela et al., 2006; Sghaier et al., 2014). This observation supports the hypothesis that not only the reproductive output, but also the reproductive success of *P. oceanica*, has increased, probably in response to changing environmental conditions. More importantly, the repeated recruitment events recorded in four localities of the Western Mediterranean, Island of Favignana, Island of Ustica, Gulf of Ventilegne and Livorno, demonstrate the existence of areas particularly favorable to the establishment of seedling populations. The first two localities are located along the Sicilian coasts, in the center of a biogeographic transition zone separating the western and eastern Mediterranean basins (Bianchi, 2007). Studies have shown that many meadows present along these coasts have flowered almost every year from 1978 to 2005 and produced large amounts of seeds (Calvo et al., 2010). Past studies suggested that the warmer south Mediterranean waters of Sicily are the best environment for *P. oceanica* flowering and growth, while the winter seawater temperatures of the northwestern Mediterranean are below the optimum for flowering induction (Molinier and Picard, 1952; Semroud, 1993). However, recent studies indicated that temperature variation rather than mean value could be the main trigger of flowering (Diaz-Almela et al., 2007). Since the genetic diversity of some meadows in the biogeographic transition zone appears be higher compared to others meadows of the Mediterranean (Jahnke et al., 2015), future studies should investigate whether there is a relationship between the success of seed production in these meadows and their genetic diversity. Flowering had rarely been observed before 1984 along the coasts of Corsica and Livorno (Caye and Meinesz, 1984), but from 1994 onward this phenomenon occurred regularly (Balestri, 2004; Gobert et al., 2005), possibly due to moderate warming of the northwestern Mediterranean over the past decades (Lejeusne et al., 2010; Marbà et al., 2015). Given the extremely high number of seedlings recruited in the Gulf of Ventilegne (up to 480 seedlings m^-2^, Balestri and Lardicci, 2008) and their persistence and growth, this site presently has a high prospect for supporting the development of newly established clones in the future. Cleary, a systematic longer-term monitoring of recruitment sites is needed to confirm the hypothesis.

### Habitat Type, Depth, and Colonization Success

The presence of recruits established on new unoccupied substrate areas at very shallow depths supports the hypothesis that sexual recruitment within established meadows is a highly improbable event for *P. oceanica* accordingly to the initial seedling recruitment model (Arnaud-Haond et al., 2014). The finding of seedlings mainly in sheltered shallow sites suggests that coastal morphology and in particular the presence of wave-sheltered inlets, such as bays and gulfs, along the fruit dispersal trajectory could play a key role in determining the initial distribution of seedlings, capturing and retaining most floating fruits until seed release. A similar apparent “preference” for sheltered shallow sites has been reported for the congeneric Australian species, *Posidonia australis* Hook. f. (Meehan and West, 2004). The presence of *P. oceanica* seedlings at shallow (0–3 m) depth indicates that neither photo-damage nor high hydrodynamic forces and temperatures generally associated with shallow depths may affect seedling establishment, at least its initial stages. Previous studies have shown that a low light environment is more favorable to *P. oceanica* growth than a high light one, and that plants growing in the upper stands of meadows (<5 m depth) are under stressful light conditions, but physiologically adapted to these conditions and genetically differentiated from those living in deeper stands (Dattolo et al., 2014). However, other studies have found that seedlings require an environment with relatively higher light compared with adults, an adaptation strategy to survive the oligotrophic conditions of the Mediterranean (Celdrán and Marín, 2013). This strategy could explain the presence of seedlings in very shallow areas as well as their apparent absence at greater depths or inside well-established stands. On the other hand, at shallow depths near-bottom orbital velocities are too high also for adult shoot establishment except in very sheltered conditions (Infantes et al., 2009). As seedlings have been found to be vulnerable to physical perturbations related to wave and currents (Infantes et al., 2011), prolonged periods of calm sea conditions could be required for the establishment of seedlings at shallow depths. Historically, *P. oceanica* has been considered a species that grows preferably on sandy habitats, although a recent study (Montefalcone et al., 2016) has shown the presence of meadows developed on rocks at very shallow depths (1.5 m depth), outside the theoretical reference zone for the meadow upper limit (>5 m depth) predicted by models (Vacchi et al., 2014). The capacity of seedlings to establish on both rocky and sandy substrates could be linked to the plasticity of the root system (Balestri et al., 2015) and to the production of adhesive root hairs on rocky substrate in the early phase of their development (Badalamenti et al., 2015). But other factors, such as the presence of algal vegetation and fissures on rocky substrates, could also play a role facilitating seedling anchorage (Alagna et al., 2013; Balestri et al., 2015). However, previous studies reported higher mortality rates of seedlings on sandy patches than on rock during their first 2–3 years. This is probably because the spatial extension of the root system of seedlings is not enough to counteract hydrodynamic forces generated by stronger storms at shallow depths on unstable substrates. Results of our analysis showed that the chance of survival and formation of new clones was effectively lower for seedlings established on sandy habitats than those on rocky ones irrespective of depth, reinforcing the hypothesis that substrate stability or complexity could ultimately determine the colonization success of the species (Balestri et al., 1998b; Badalamenti et al., 2015).

### Ecological and Conservation Implications

The ability of seedlings of *P. oceanica* to grow and establish new patches outside the upper limit of extant meadows and the recurrent occurrence of sexual reproductive events highlight the importance of developing additional management measures to improve the effectiveness of habitat/species conservation in the future. Such measures could include, for example, actions for the protection of those meadows that flower more profusely to ensure the conditions required for the development and ripening of seeds, as well as the development of programs specifically designed to identify, monitor and protect new recruitment localities. In fact, multiple strategies have been attempted in many European countries to preserve *P. oceanica* meadows in the face of predicted climate-induced alterations. However, monitoring programs have focused on well-established meadows and most monitoring stations are currently located in the shallow- or mid depth of meadows, usually below 5 m of depth (Montefalcone, 2009; Boudouresque et al., 2012), thus outside the present range of distribution of established seedlings. Moreover, some conservation efforts have attempted to improve coastal seawater quality and remove disturbances that have caused or will potentially lead to the decline of existing meadows (EU Water Framework Directive, WFD, 2000/60/EEC, Marine Strategy Framework Directive MSFD, 2008/56/EEC). The rationale governing these efforts is to maintain the environmental services provided by meadows, which are directly or indirectly related to surface and cover of well-established meadows. However, populations of long-lived species dominated by a few old clones, such as *P. oceanica*, may persist despite suboptimal site conditions, and thus their patch occupancy pattern may not be in equilibrium with the present landscape configuration but rather reflect the historical landscape (Eriksson, 1994). Even occasional inputs of new genets may increase the probability of adaptation-capacity of species to changing environmental conditions (Ehlers et al., 2008; Hughes and Stachowicz, 2009).

Projections indicate that the sea level in Mediterranean will rise over 30–60 cm in the next century (IPCC, 2007). For example, in Corsica, the mean sea level has risen by 18 cm between 1870 and 2000, including 6 cm over the past 20 years, causing the regression of some *P. oceanica* meadows near the depth of compensation of the species and in sectors where the slope is relatively slight (Pergent et al., 2015). Since the prevalent trajectory of colonization of the seedlings in Corsica appears to be toward the coastline, predicted sea level rise could alleviate environmental stresses (physical disturbances, photo-damage, and temperature fluctuations) experienced by surviving seedlings, and provide an opportunity for the species to move landward and colonize newly submerged areas. Moreover, results or recent laboratory studies suggested that projected increases in *p*C0 _2_ concentrations could have positive effects on seedlings by increasing their photosynthetic performance and carbon fixation activity during the initial phases of development, enabling them to better resist or recover from stressful conditions, like adverse temperature and light conditions (Hernán et al., 2016). However, stronger storms, anthropogenic pressure, coastal activities and presence of physical barriers in the vicinity of recruitment sites, could be important threats for seedlings. Fortunately, many recruits such as those observed in Corsica were located inside the boundaries of marine protected areas or national parks. Nevertheless, damage to established meadows due to recreational activities (fishing, boat anchoring, sewage effluents or organic inputs from visitors) has been reported in some protected areas (Marbà et al., 2002; Bonacorsi et al., 2013). More restrictive measures would therefore be necessary to reduce the potential impact of such activities on seedlings, in order to facilitate their expansion on new available sites. In exposed sites, it could also be necessary to use anchoring and protective structures for seedlings to reduce the risk of plant dislodgment during storms as well as to prevent overgrowth of out-competing macro-algae.

Seedlings are increasingly an important management tool for seagrass restoration, and studies have shown that cultivated seedlings of *P. oceanica* can be transplanted in the field with relatively high success and thus used as planting material for assisted colonization at shallow sites or for restoration of small damaged areas in disturbed meadows (Balestri et al., 1998b; Domínguez et al., 2012; Terrados et al., 2013). Since stranded *P. oceanica* fruits are increasingly available in many countries, they may be planted in selected suitable areas to create reservoirs of juveniles for future restoration activities. Temporary nurseries could be also created into aquaculture facilities for the duration of restoration programs (Balestri and Lardicci, 2012; Leal et al., 2016). The harvesting of seedlings in natural recruitment sites and their translocation into distant recipient sites should be not encouraged.

## Conclusion

Our analysis of recorded recruitment events, along with the identification of four regeneration sites in the Western Mediterranean that consistently supported the establishment of seedlings, contradicts the historical assumption that sexual recruitment is a sporadic and unsuccessful event for *P. oceanica*. Recruitment sites shared some similar environmental characteristics, i.e., located in sheltered areas (inlets, bays, and gulfs), prevalent on protected zones situated along the coasts of islands and at very shallow depths. More importantly, our 11 years of observations document for the first time the formations and development of clonal patches of *P. oceanica* by seed. Notably, most recruitment sites are near established meadows but at shallower depths than their upper limit, and thus ignored by monitoring programs. Successful recruit inputs in shallow unoccupied areas could give the species a chance to establish new genets and expand landward under sea-level rise scenarios, in the absence of physical barriers and anthropogenic pressures. Instead, recruitment on dead matte areas could play a role in the recovery of meadows after small scale disturbances. Given the potential importance of new genetic inputs for the long-term viability of the species and persistence in changing coastal environmental conditions, a more integrated conservation approach to include the protection of areas that presently support or that are expected to support new seedling populations in the future will be necessary.

## Author Contributions

EB: designed the work, analyzed, interpreted seedling data for the work; and drafted the work; and approved the final version to be published; and agreed to be accountable for all aspects of the work in ensuring that questions related to the accuracy or integrity of any part of the work are appropriately investigated and resolved. FV: acquisited, analyzed seedling data for the work; and revised critically for important intellectual content; and approved the final version to be published; and agreed to be accountable for all aspects of the work in ensuring that questions related to the accuracy or integrity of any part of the work are appropriately investigated and resolved. CL: participated to design the work, interpreted seedling data for the work; and revised it critically for important intellectual content; and approved the final version to be published; and agreed to be accountable for all aspects of the work in ensuring that questions related to the accuracy or integrity of any part of the work are appropriately investigated and resolved.

## Conflict of Interest Statement

The authors declare that the research was conducted in the absence of any commercial or financial relationships that could be construed as a potential conflict of interest.
